# Mechanisms underlying clinical efficacy of Angiotensin II type 2 receptor (AT_2_R) antagonist EMA401 in neuropathic pain: clinical tissue and in vitro studies

**DOI:** 10.1186/s12990-015-0038-x

**Published:** 2015-06-26

**Authors:** Uma Anand, Yiangos Yiangou, Marco Sinisi, Michael Fox, Anthony MacQuillan, Tom Quick, Yuri E Korchev, Chas Bountra, Tom McCarthy, Praveen Anand

**Affiliations:** Peripheral Neuropathy Unit, Centre for Clinical Translation, Hammersmith Hospital, Imperial College London, Area A, Ground Floor, Du Cane Rd, London, W12 ONN UK; Nanomedicine Research Laboratory, Division of Medicine, Hammersmith Hospital, Imperial College London, BN5 Commonwealth Building, London, W12 0NN UK; Peripheral Nerve Injury Unit, Royal National Orthopaedic Hospital, Stanmore, Middlesex HA7 4LP UK; Spinifex Pharmaceuticals Pty Ltd, Corporate One, Suite G5, 84 Hotham St, Preston, VIC 3072 Australia; University of Oxford Structural Genomics Consortium, Old Road, Campus Research Building, Roosevelt Drive, Headington, Oxford, OX3 7DQ UK

**Keywords:** Angiotensin II, AngIII, Ang-(1-7), AT_2_R, Antagonist, EMA401, Human DRG neurons, Peripheral nerve injury, Calcium imaging, Hypersensitivity, Neuropathic pain, Neurites

## Abstract

**Background:**

The clinical efficacy of the Angiotensin II (AngII) receptor AT_2_R antagonist EMA401, a novel peripherally-restricted analgesic, was reported recently in post-herpetic neuralgia. While previous studies have shown that AT_2_R is expressed by nociceptors in human DRG (hDRG), and that EMA401 inhibits capsaicin responses in cultured hDRG neurons, the expression and levels of its endogenous ligands AngII and AngIII in clinical neuropathic pain tissues, and their signalling pathways, require investigation. We have immunostained AngII, AT_2_R and the capsaicin receptor TRPV1 in control post-mortem and avulsion injured hDRG, control and injured human nerves, and in cultured hDRG neurons. AngII, AngIII, and Ang-(1-7) levels were quantified by ELISA. The in vitro effects of AngII, AT_2_R agonist C21, and Nerve growth factor (NGF) were measured on neurite lengths; AngII, NGF and EMA401 effects on expression of p38 and p42/44 MAPK were measured using quantitative immunofluorescence, and on capsaicin responses using calcium imaging.

**Results:**

AngII immunostaining was observed in approximately 75% of small/medium diameter neurons in control (n = 5) and avulsion injured (n = 8) hDRG, but not large neurons i.e. similar to TRPV1. AngII was co-localised with AT_2_R and TRPV1 in hDRG and in vitro. AngII staining by image analysis showed no significant difference between control (n = 12) and injured (n = 13) human nerves. AngII levels by ELISA were also similar in control human nerves (4.09 ± 0.36 pmol/g, n = 31), injured nerves (3.99 ± 0.79 pmol/g, n = 7), and painful neuromas (3.43 ± 0.73 pmol/g, n = 12); AngIII and Ang-(1-7) levels were undetectable (<0.03 and 0.05 pmol/g respectively). Neurite lengths were significantly increased in the presence of NGF, AngII and C21 in cultured DRG neurons. AngII and, as expected, NGF significantly increased signal intensity of p38 and p42/44 MAPK, which was reversed by EMA401. AngII mediated sensitization of capsaicin responses was not observed in the presence of MAP kinase inhibitor PD98059, and the kinase inhibitor staurosporine.

**Conclusion:**

The major AT_2_R ligand in human peripheral nerves is AngII, and its levels are maintained in injured nerves. EMA401 may act on paracrine/autocrine mechanisms at peripheral nerve terminals, or intracrine mechanisms, to reduce neuropathic pain signalling in AngII/NGF/TRPV1-convergent pathways.

## Background

Neuropathic pain has a prevalence of 1–8% in the general population, and is a major unmet medical need due to limited efficacy and tolerability of currently available drug treatments. Hyperexcitability and abnormal sprouting of primary afferent sensory nerve fibres underpin peripheral neuropathic pain [1].

The angiotensin II (AngII) type 2 receptor (AT_2_R) was identified as a novel target for neuropathic pain: several small molecule AT_2_R antagonists with >1000-fold selectivity over the AT_1_R receptor produced dose-dependent analgesia in multiple rodent neuropathic pain models, with the analgesia abolished in mice null for the AT_2_R [2]. Our studies in human and rat DRG neurons demonstrated that EMA401, a selective AT_2_R antagonist, inhibited neurite outgrowth and capsaicin responses in cultured human and rat DRG neurons, in an in vitro model of sensitization [3]. In a recent randomized, double-blind, placebo-controlled clinical trial in patients with post-herpetic neuralgia, administration of the orally active AT_2_R antagonist, EMA401, for 4 weeks produced significant pain relief above placebo and was well-tolerated [4].

The underlying mechanisms of AT_2_R signalling in clinical neuropathic pain states, and mode of action of the peripherally-restricted AT_2_R antagonist EMA401, require elucidation. We have previously shown that AngII, the endogenous ligand for the AT_2_R, acted in vitro on AT_2_R resulting in sensitization of TRPV1 and increased neurite outgrowth in DRG neurons via increased cAMP, which were both inhibited in the presence of EMA401 [3]. TRPV1, the heat and capsaicin receptor, is known to be sensitized when phosphorylated by cAMP, and desensitized when dephosphorylated [5]. Neurite outgrowth is also sensitive to cAMP levels, and our previous study showed the neurite promoting effects of AngII in the presence of added neurotrophic factors, which have similar effects [3]. In animal models of neuropathic pain the analgesic action of AT_2_R antagonists involves inhibition of enhanced AngII/AT_2_R induced p38 and p42/p44 MAPK activation [2].

Systemic AngII is known to be derived from the action of kidney derived renin on AngI in the circulation. However, local renin angiotensin system (RAS) components have been described in brain [6], heart, blood vessels [7], and DRG [8, 9], constituting potential local sources of AngII, separate from the circulating AngII. In addition to AngII, other components of the RAS, AngIII and Ang-(1-7), are also ligands at the AT_2_R; however the levels of these endogenous ligands in clinical peripheral neuropathic tissues and signalling pathways are unknown. AngII to AngIII conversion in rodent CNS has been reported to mediate descending inhibitory pathways, while Ang-(1-7) is a biologically active heptapeptide formed endogenously from either AngI or AngII, with vasodilatory and antiproliferative activities that oppose the constrictive and proliferative effects of AngII [10, 11]. Circulating levels of Ang-(1-7) in humans are reported to increase following long term administration of ACE and AT_1_ receptor blockers [12–14].

We have studied the expression of AngII, AT_2_R and TRPV1 in clinical tissues, and quantified the levels of AngII, AngIII and Ang-(1-7) by ELISA. The effects of AngII, the AT_2_R antagonist EMA401 and the synthetic AngII agonist EMA1087 (Compound **21**) [15] on calcium influx and neurite outgrowth were studied in cultured rat DRG neurons. The in vitro effects of AngII, NGF and EMA401 on expression of pp38 and pp42/44 MAPK in rat sensory neurons were measured using immunofluorescence. AngII, AT_2_R and TRPV1 expression were also studied in cultured human DRG neurons using immunofluorescence, and signalling pathways using calcium imaging.

## Results

### AngII antibody characterisation

#### Immunohistology

**Figure 1:**
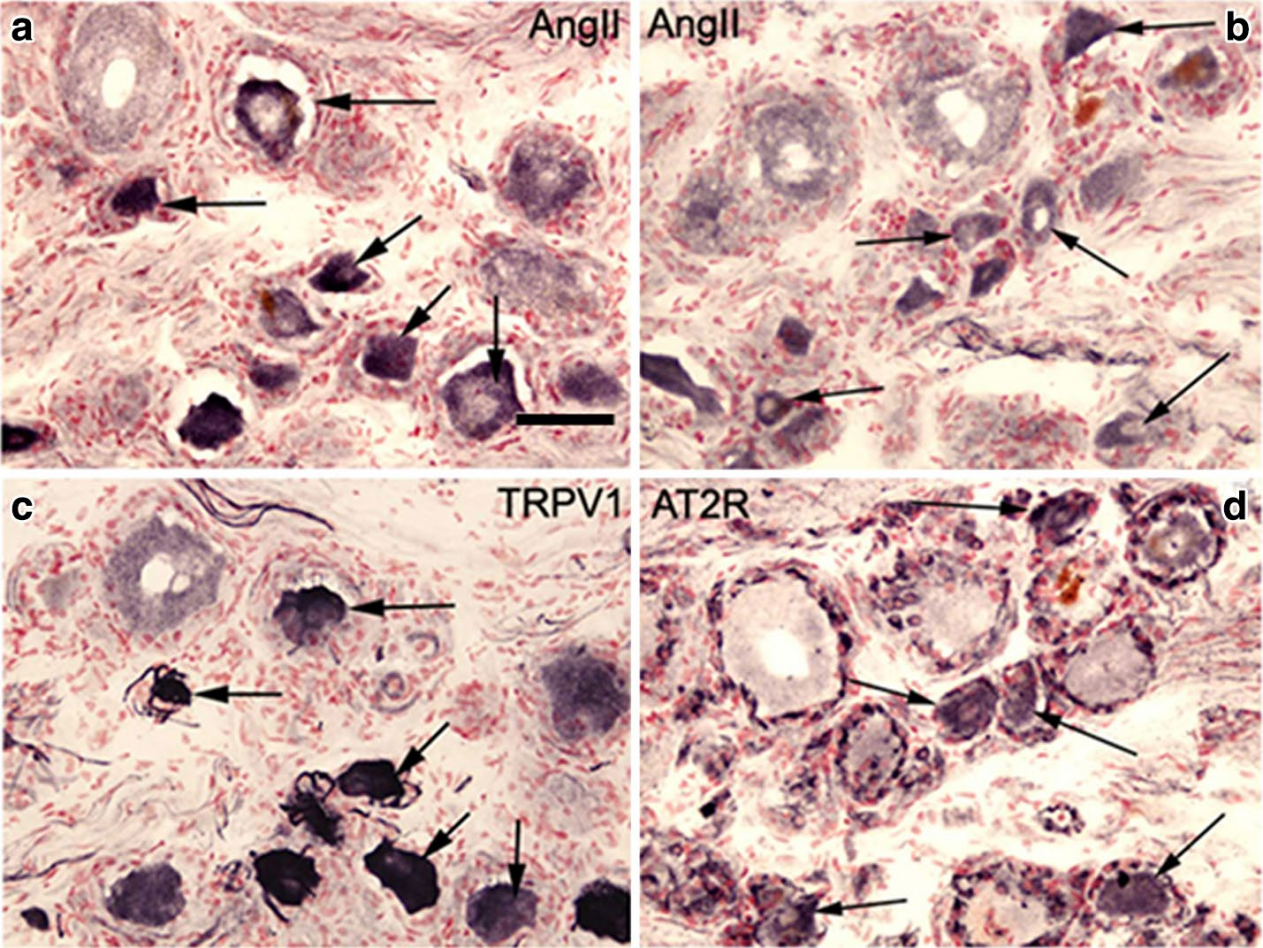
IHC in human DRG tissues. Serial sections of post-fixed human avulsion injured DRG immunostained with rabbit antibodies to AngII (**a**) and TRPV1 (**c**). *Arrows* indicate co-localising cells. Similar serial sections of post-fixed human avulsion injured DRG immunostained with antibodies to AngII (**b**) and AT_2_R (**d**). *Arrows* indicate co-localising cells. *Scale bar* 50 microns.

All results are given as mean ± sem unless otherwise stated. Antibodies to AngII (Table 1) were first evaluated by titration on tissue sections of human DRG. Rabbit antibodies to AngII showed strong staining of small/medium diameter (≤50 µm) sensory neurons but not large diameter neurons (Figure 1a, b), and showed co-localisation with TRPV1 (Figure 1c). Mouse antibodies showed very similar strongly immunoreactive sensory neurons (not shown). Counts of AngII immunoreactive small/medium diameter (≤50 µm) neurons showed that 78.54 ± 2.0% were positive in control hDRG (n = 5) and 72.13 ± 5.4% in avulsion injured hDRG (n = 8, p = 0.95). A comparison of serial sections immunostained with AngII (Figure 1b) and AT_2_R antibody showed that AngII and AT_2_R were co-localised in some small/medium diameter (≤50 µm) cells (Figure 1d).

**Table 1:** Antibodies used for immunostaining human tissues

Antibody	Donor	Source	Reference	Dilution
AngII/III	Mouse	Serotec Ltd. UK	0500–0300	25–1,000
AngII	Rabbit	BIOSS-USA	bs-0587R	5–1,000
TRPV1	Rabbit	GSK	C22	10,000
AT2 receptor	Goat	Santa Cruz	sc-48452	50–100

**Figure 2:**
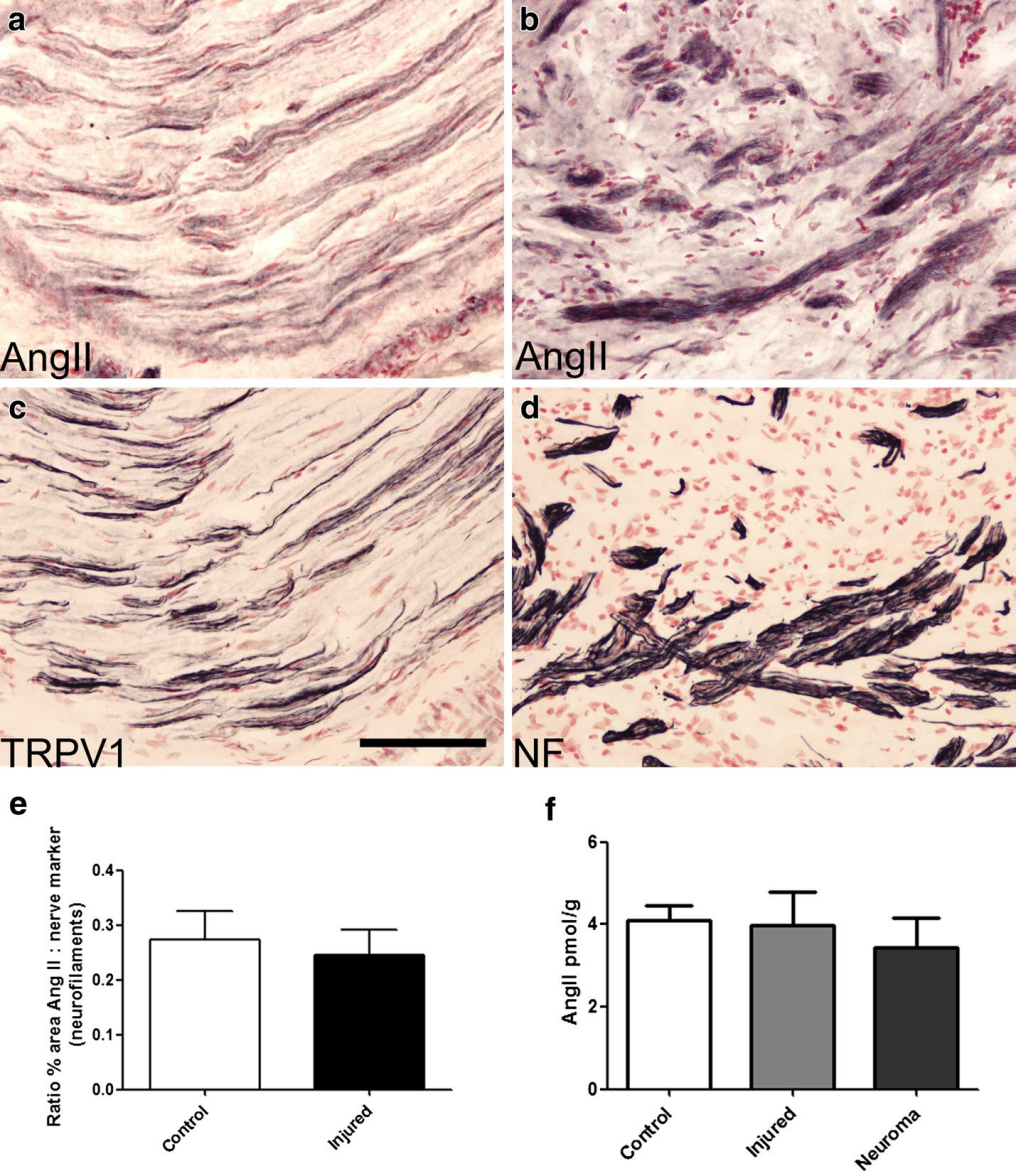
IHC in human nerve tissue. Serial sections of post-fixed human peripheral nerve immunostained with antibodies to AngII (**a**, **b**) showing co-localisation with TRPV1 (**c**) and the structural nerve marker Neurofilament (NF) (**d**). *Scale bar* 100 microns. Graph showing image analysis (% area) of AngII immunoreactivity in control (n = 12) and injured (n = 13) nerves expressed as a ratio to the structural nerve marker neurofilaments (**e**). Graph showing AngII levels by ELISA were similar in control human nerves (n = 31), injured human nerve trunks (n = 7), and painful human neuromas (n = 12) (**f**).

Serial sections of human peripheral nerve immunostained with antibodies to AngII (Figure 2a), and TRPV1 (Figure 2c) showed positive nerve fibres. AngII was present in injured human nerve fibres (Figure 2b), also shown with the structural nerve marker neurofilaments (Figure 2d). Image analysis (% area) of nerves immunostained with AngII antibodies showed no significant difference between control (n = 12) and injured (n = 13) human nerves (Figure 2e). AngII levels by ELISA were also similar in control human nerves (4.09 ± 0.36 pmol/g, n = 31), injured nerve trunks (3.99 ± 0.79 pmol/g, n = 7), and painful neuromas (3.43 ± 0.73 pmol/g, n = 12) (Figure 2f). AngIII-specific and Ang-(1-7) ELISA levels were undetectable (below <0.03 and <0.05 pmol/g respectively), indicating that the major angiotensin analogue in these human nerve tissue extracts is AngII.

#### Results of in vitro studies

**Figure 3:**
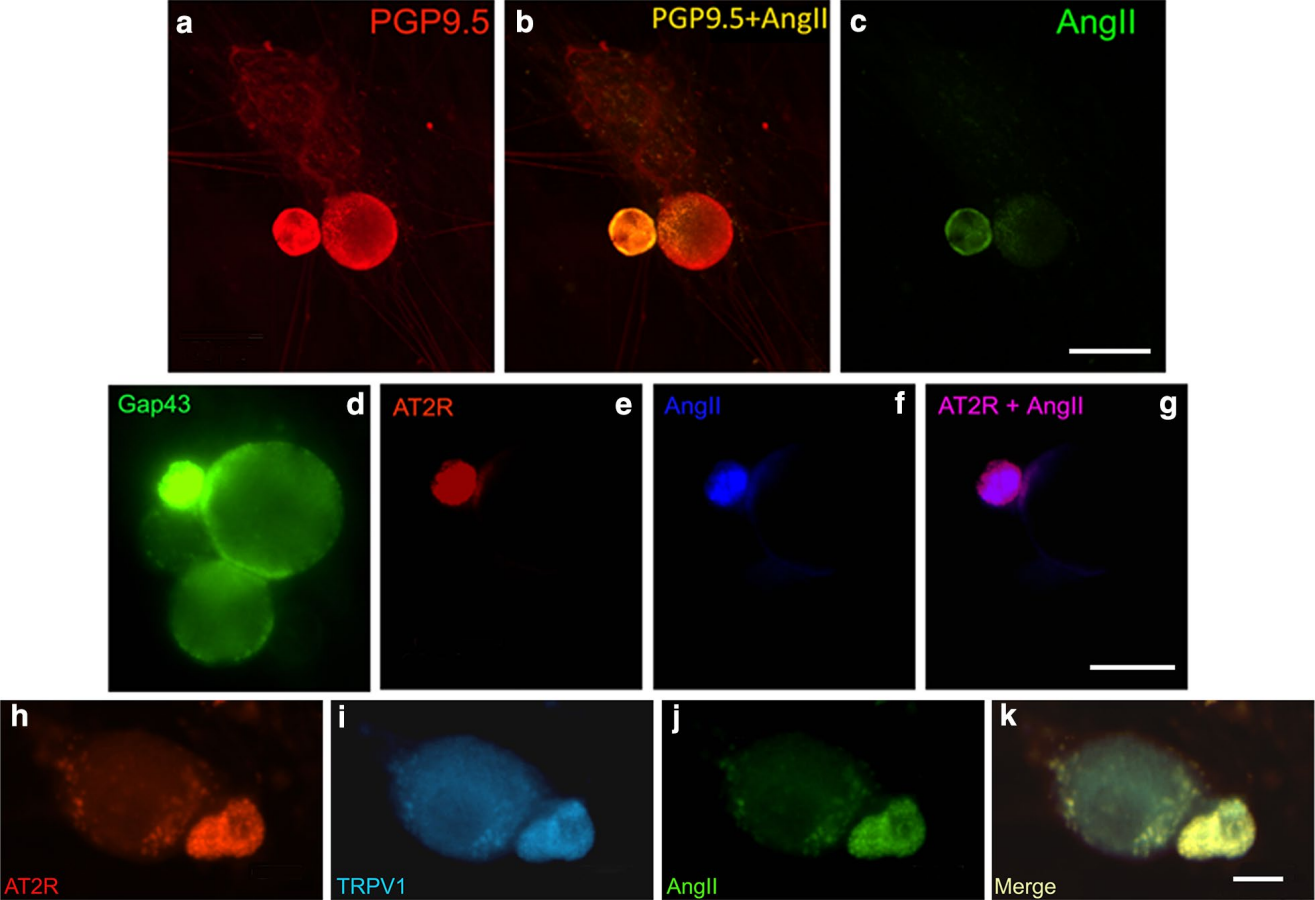
Immunofluorescence in cultured hDRG neurons. IF images of cultured hDRG neurons positive for the neuronal marker PGP9.5 (**a**, *red*), co-localised with AngII expression in small diameter neuron (*yellow in merged image*, **b**), but not in large neuron, and AngII expression alone (**c**, *green*). *Bar* 50 μM. IF images showing co-localization of Gap43 (*green*, **d**), AT_2_R (*red*, **e**), and AngII (*blue*, **f**) in small diameter cultured hDRG neurons; merged AT_2_R and AngII (**g**). *Bar*  20 μm. Co-localization of AT2R (*red*, **h**), TRPV1 (*blue*, **i**), AngII (*green*, **j**) and the merged image (**k**). *Bar* 10 μm.

**Figure 4:**
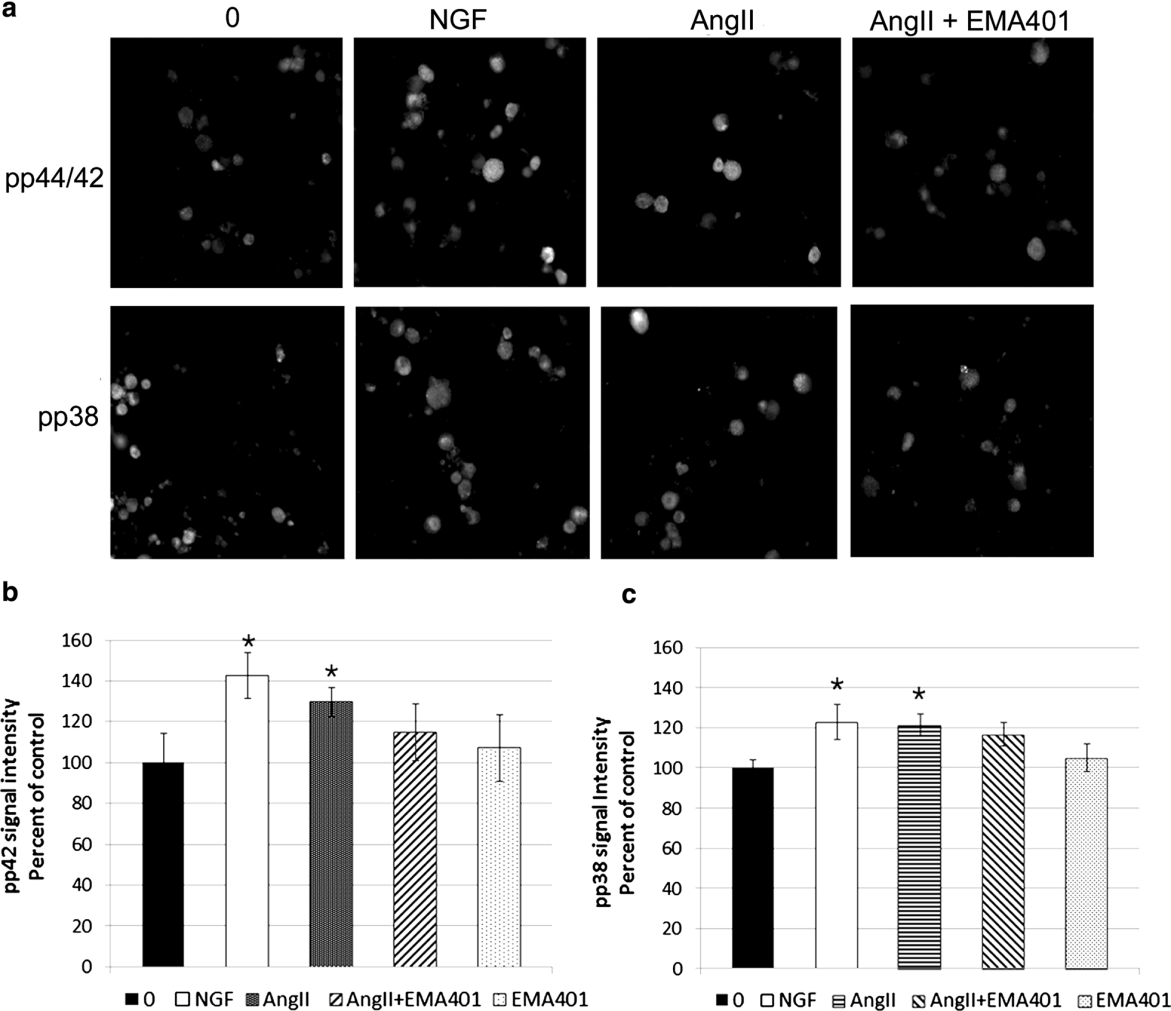
pp42/44 and pp38 expression in cultured rDRG neurons. IF images of pp42/44 expression (*upper panel*), and pp38 expression (*lower panel*) in vehicle treated rDRG neurons (control 0), or treated with NGF, AngII, or AngII + EMA401 (**a**). Graph showing quantitation of signal intensity for pp42/44 (**b**), and pp38 expression (**c**).

These studies showed co-expression of AT_2_R, AngII and TRPV1 in small diameter cultured hDRG neurons using immunofluorescence (Figure 3). AngII was expressed in 75.6 ± 6.3% small diameter neurons (≤50 μm diameter, 509 neurons), with a mean diameter of 37.5 ± 1.8 μm, and co-localised with virtually all AT_2_R positive hDRG neurons. AngII was co-localised in cultured DRG neurons with AT2R and TRPV1, as illustrated in Figure 3h–k. AngII treated rDRG neurons showed a significant increase in pp42/44 signal intensity compared to vehicle treated controls (*P < 0.05), similar to the positive controls treated with NGF (*P < 0.05, Figure 4). Signal intensity was reduced to an extent in cultures treated with AngII combined with EMA401 (P > 0.05). Similar increases in pp38 signal intensity were observed in NGF (*P < 0.05) and AngII treated neurons (*P < 0.05), which were reduced to an extent after co-incubation with EMA401 (P > 0.5, Figure 4).

**Figure 5:**
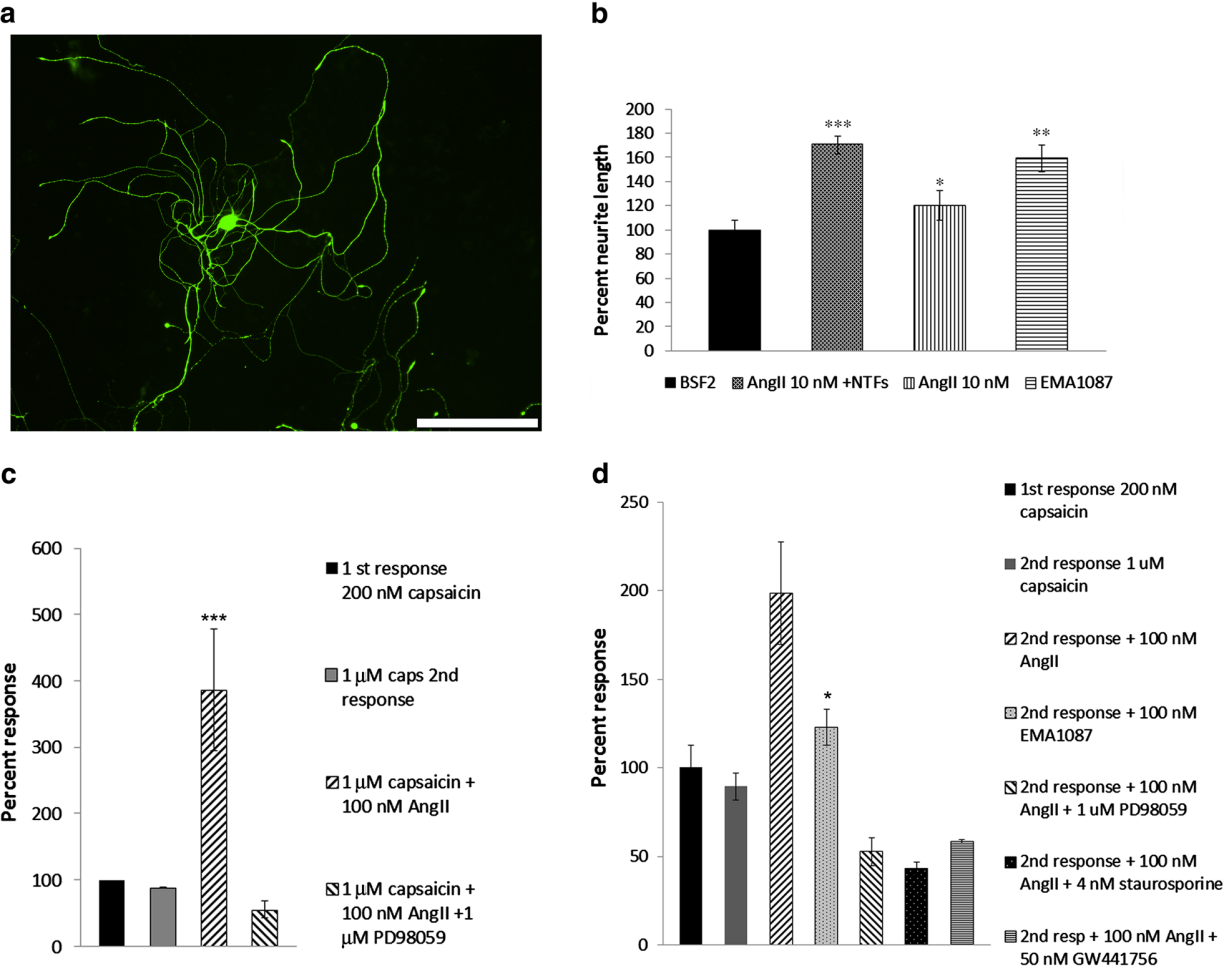
Effect on neurite length and calcium imaging. Image of an AngII treated DRG neuron showing β tubulin immunofluorescent cell soma and neurites (**a**), *bar* 200 μm. Graph showing comparative neurite lengths of neurons treated with BSF2 (medium alone), and significant increase with AngII + NTFs, Ang II or EMA1087 (**b**). AngII mediated sensitization of hDRG neurons was reversed in the presence of the MAPK inhibitor PD98059 (**c**). The commercial AT_2_R agonist EMA1087 (Compound **21**) also caused significant sensitization of capsaicin responses. AngII mediated sensitization was abolished in the presence of PD98059, staurosporine, and TrkA inhibitor GW441756 (**d**).

The average neurite lengths from each group were normalised to vehicle treated controls (BSF2 medium); neurite lengths were significantly increased in rDRG neurons treated with combined AngII and NTFs (170.6 ± 7.3%, ***P < 0.001, n = 3), or AngII alone (120.5 ± 12%, *P < 0.05, n = 4), or EMA1087 (C21) alone (159.2 ± 11%, **P < 0.01, n = 4), compared with vehicle treated controls (694.7 ± 55 μm, n = 5), (Figure 5a, b).

Calcium imaging showed that in hDRG, the second response to capsaicin (control) in the absence of added drugs was reduced due to repeat stimulation (tachyphyllaxis) (83.1 ± 2.3%, n = 23). Capsaicin responses were significantly enhanced in the presence of 100 nM AngII, compared with responses in the absence of AngII (n = 4, ***P < 0.01); this enhancement was not observed in the presence of the MAPK inhibitor PD98059 (1 μM, n = 8) (Figure 5c). Similarly, the second response (control) in rDRG neurons was reduced to 89.5 ± 7.7% (n = 14), but showed significant enhancement of capsaicin responses in the presence of AngII (198.5 ± 29%, n = 16, ***P < 0.001), and EMA1087 (C21, 123.2 ± 10%, n = 4, *P = 0.013). While rDRG neurons also showed sensitization after AngII treatment, the magnitude was less than that observed in hDRG neurons. AngII mediated sensitization was not observed in combination with PD98059 (n = 9 neurons), the protein kinase inhibitor staurosporine (n = 11 neurons), and the TrkA inhibitor GW441756 (n = 12 neurons), (Figure 5d).

**Figure 6:**
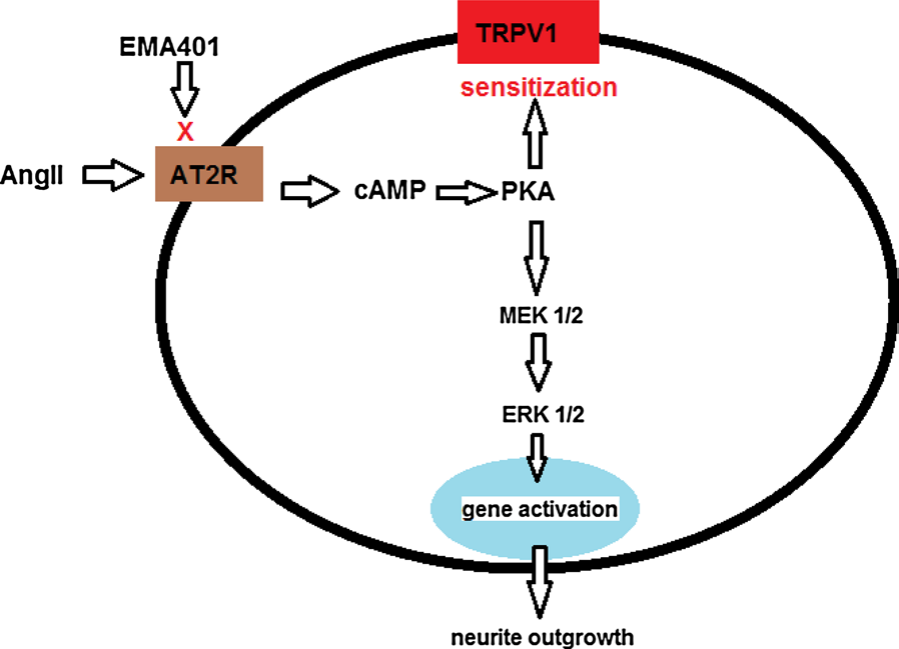
Diagram indicating the pathway involved in AngII and TRPV1 signalling, with activation of p38 (MAPK) and p44/42 (ERK) and their inhibition by EMA401 in DRG neurons.

A schematic diagram showing the signalling pathway involving AT2R and TRPV1 is shown in Figure 6.

## Discussion

In this study we observed that AngII was expressed in a large proportion of small diameter neurons in human DRG, co-localised with AT_2_R and TRPV1, and that AngII levels were preserved in injured human DRG and nerve tissues. Co-localisation of AngII expression with AT_2_R in hDRG, and its levels in injured nerves, supports the concept of an intrinsic neuronal angiotensinergic system, and suggests that EMA401 may act via autocrine/intracrine in addition to paracrine mechanisms in DRG neurons, to reduce augmented neuropathic pain signalling.

Quantitative ELISA studies in human tissues showed that the major AT_2_R ligand in human peripheral nerves was AngII, and that its levels were similar in injured human nerves and painful neuromas compared to control nerves. Evidence supports the concept of an intrinsic neuronal angiotensinergic system, with intraneuronal AngII formation in sensory neurons, which appears to be maintained in injured neurons—hence its potential autocrine/intracrine role in pathological nociceptive mechanisms. AngIII and Ang-(1-7) levels were below the detection limits, in accord with other studies [8]. Previous studies have also described dense AngII positive nerve fibres and high levels in peripheral organs [16], and localisation in small and medium sized neurons in trigeminal ganglia [8, 9]. In situ hybridization in rat trigeminal ganglia have revealed expression of AngII precursor Angiotensinogen (Ang-N) mRNA in the cytoplasm of numerous neurons, and post in situ hybridization immunocytochemistry marked some of these for Ang II; substance P was found colocalized with Ang II [8]. These studies support intraneuronal AngII formation in sensory neurons, separate from circulating AngII, and its potential autocrine/intracrine role in nociceptive mechanisms.

It is important to note that we have measured AngII levels in human nerves and DRG by ELISA in frozen tissue extracts; as AngII may degrade when extracted in frozen tissues, freshly extracted tissues need to be studied in future for AngII, other putative endogenous RAS ligands, and their metabolites. The levels obtained in our study are higher than those reported in other studies of rat and human trigeminal ganglia [8, 9], and may reflect differences in peptide extraction prior to assay.

Injury and associated inflammation may increase local AngII, contributing to sensitization of nociceptors, particularly in sprouting nerve fibres. The molecular regulation of AT_2_R and AngII expression in injured human or rodent sensory neurons is unknown, and may be influenced by target-organ derived or intrinsic factors. In our previous study [3], AT_2_R levels were reduced in human nerve segments proximal to injury (lesion distal to the DRG, or ‘peripheral axotomy’), but they were preserved or high in chronic painful neuromas, which comprise regenerating nerve fibres. These AT_2_R receptor level changes parallel those of key pain receptors and ion channels regulated by target-derived growth factors e.g. TRPV1, Nav 1.7 and Nav1.8 by NGF after nerve injury. The mode of analgesic action of EMA401 may involve inhibition of augmented AngII/AT_2_R signalling in NGF convergent pathways, a postulate which is supported by our in vitro results, described below. The lack of CNS penetration of EMA401, and the time-course of its clinical effects in patients with post-herpetic neuralgia (i.e. gradually progressive over 4 weeks, unlike anticonvulsants) argue against AngII acting as a classical neurotransmitter in dorsal spinal cord.

Co-expression of AngII, AT2R and TRPV1 was observed in cultured small diameter hDRG neurons using immunofluorescence, in accord with the hDRG tissue findings. AngII was co-localised in cultured DRG neurons with AT2R and TRPV1, as illustrated in Figure 3h–k. In AngII treated rat neuronal cultures we observed significantly increased levels of pp38 and pp42, similar to the effect of NGF, which were reduced in the presence of EMA401. These results indicate that AngII mediated sensitization involves p38 and p44/42 phosphorylation, and that EMA401 is effective in blocking this in vitro, in agreement with animal models of pain [2, 17]. It appears that the mechanism of AngII mediated p38 and p44/42 phosphorylation involves interaction of the AT_2_R and TrkA, which underlies neuronal differentiation of NG108 cells [18], sensitization of mature neurons as observed in this study, and increased neurite outgrowth.

In our previous study [3], neurons treated with combined AngII and NTFs (NGF, GDNF, NT3) had significantly longer neurites compared with NTF treatment alone, suggesting a synergistic effect of AngII with the neurotrophic factors, which was reduced by co-treatment with EMA401. In this study neurite lengths were also significantly increased in neurons treated with AngII and EMA1087 (C21) alone, but less than with added NTFs. The effect of EMA401 on neurites was diminished outgrowth, rather than degeneration of established neurites. Thus AngII treatment of DRG neurons leads to increased neurite length and TRPV1 sensitization by increased cAMP, both of which are blocked by EMA401 [3]. While AT_2_R activation appears to have a synergistic effect with TrkA, other pathways such as PI3K [19] may also be involved, and need investigation. Other studies have reported a role for the AT_2_R in mediating neurite outgrowth in vitro via estrogen activation [20, 21], and in a rodent model of inflammatory hypersensitivity [22]. These mechanisms may contribute to the analgesic effects of AT_2_R antagonists in such animal models, and in patients with chronic pain, particularly those more prevalent in females.

Our finding that AngII and EMA1087 (C21), a small molecule AT_2_R agonist, caused neurite outgrowth in our assay is in agreement with the neurite promoting effects of C21 previously described in the neuroblastoma-glioma hybrid NG108 cells, that was blocked by the AT_2_R receptor antagonist (PD-123,319) [15, 23]. PD123,319 was effective in reducing neurite growth in a CFA inflammatory pain rodent model [22], and EMA401 inhibited neurite outgrowth in vitro [3], confirming their antagonist effects at the AT_2_ receptor, and potential efficacy in pain states associated with abnormal nerve sprouting. Conceptually, while promoting AT_2_ receptor mediated neuronal outgrowth could be beneficial after certain pathologies (e.g. stroke), aberrant or collateral neuronal regeneration with hypersensitivity may underpin some painful conditions in the periphery mediated via AT_2_R [22], in accord with our clinical efficacy data with EMA401 in postherpetic neuralgia patients, and human DRG nociceptor models in vitro [3, 4]. An important consideration is that the neurite promoting effects of C21 described previously in NG108 cells are derived from undifferentiated neuroblastoma-glioma hybrid cells, presented as % cells with neurites [18], with neurite extension representing neuronal differentiation of the NG108 cells, and AngII effects measured by the number of cells expressing neurites. This reflects neuronal differentiation, with C21 promoting the neuronal phenotype. The findings of our study are, however, derived from measurements of neurite lengths from mature, well differentiated neurons, and describe the effects of AngII on neurite lengths in individual neurons, relevant to hyperinnervation.

As AT_2_R is a GPCR, the AngII mediated increase in cAMP [3] is in keeping with adenylyl cyclase activation, likely to involve a G_αs_ mechanism. The consequent TRPV1 sensitization was not observed in the presence of the MAP kinase inhibitor, PD98059 in hDRG and rDRG neurons in this study. AngII effects have been associated with increased neuronal excitability and promotion of neurite outgrowth via multiple mechanisms of AT_2_R activation, depending on the cell type [19]. cAMP is also known to activate MAPK to stimulate cell growth [24], which in post-mitotic non-dividing neurons may manifest as neurite outgrowth. The increased pp38 and pp44/42 expression in AngII treated neurons indicate that AngII mediated neurite outgrowth and neuronal sensitization both involve MAPK/ERK activation, that is attenuated by EMA401. AngII thus appears to have similar effects as NGF in causing neurite outgrowth and TRPV1 sensitization [25], but not in the presence of staurosporine, PD98059 (MAPK inhibitor) and GW441756 (TrkA inhibitor).

Our results showed that both AngII and its analogue EMA1087 (C21) caused TRPV1 sensitization, suggesting that like AngII, C21 has a pro-nociceptive effect. While the effects of EMA1087 are more similar to AngII for neurite outgrowth but less so in sensitization effects, EMA1087 did show significantly enhanced responses as did AngII. EMA1087 is a synthetic molecule and AngII a peptide, which could explain differential effects in assays. AngII appeared to result in greater sensitization of human neurons compared with rat neurons, possibly reflecting a species specific difference.

Previous studies have shown pro-inflammatory effects of AngII in a variety of tissues including kidney, heart and blood vessel wall, by up-regulating the expression of both AT_1_R and AT_2_R, and activation of a number of signalling pathways including p44/42 MAPK, p38 MAPK, c-JUN and NF-κB [26, 27]. AngII and the inflammatory mediator lipopolysaccharide (LPS) are reported to upregulate the expression of AT_1_R, AT_2_R and LOX-1, and IL-1β in cardiomyocytes, with activation of MAPKs, c-Jun and NF-kB, suggesting a positive feedback between AngII and inflammation [28]. CD68 positive tissue macrophages are also reported to increase expression of angiotensinogen and renin in a model of inflammatory hypersensitivity [22]. There is thus potential for EMA401 to block a number of convergent pathways activated by a variety of inflammatory mediators. The sensitizing effect of NGF is well known [25, 29–31] and is the basis of our in vitro hDRG model of hypersensitivity, modelling the elevated NGF levels observed in tissues from chronic pain conditions [32–34]; AngII treatment caused further sensitization, suggesting functional synergy between AT_2_R and TrkA. Other pathways need investigation, such as GDNF signalling, which may have similar additive effects.

AngII has been reported to increase K^+^ channel activity in hippocampal neurons, that was reversed by the AT_2_R antagonist PD123,319 [35]. In a recent study, this mechanism of hyperpolarisation was proposed to underlie the lack of pain in Buruli ulcers caused by mycolactone, the polyketide product of *m. ulcerans* [36]. While this may reflect differences in signalling between hippocampal and DRG neurons, a number of clinical and neuropathological aspects of Buruli ulcer have drawn caution against such an interpretation [37].

## Conclusion

AngII and AT_2_R are co-expressed in nociceptive human sensory neurons, and the levels of AngII, the major endogenous ligand in human peripheral nerves, are preserved after injury. AngII induces p38, p42/p44 mitogen activated protein kinase (MAPK) activation, neurite outgrowth in adult rat DRG neurons, and sensitization of adult rat and human DRG neurons that is blocked by EMA401. Hence increased AngII/AT_2_R signalling in DRG neurons secondary to peripheral nerve injury may have a key role in chronic pain mechanisms, including neuropathic pain. The mode of EMA401 analgesic action appears to involve inhibition of augmented AngII/AT_2_R induced p38 and p42/p44 MAPK activation, and hence inhibition of DRG neuron hyperexcitability and sprouting of DRG neurons. EMA401 is likely to be most effective in conditions of hypersensitivity associated with abnormal nerve sprouting, where AngII may synergize or augment NGF mechanisms. Selective AT_2_R antagonists represent a new class of analgesics for improved relief of neuropathic pain.

## Methods

### Immunostaining for Angiotensin II (AngII) in human tissues

#### Tissues

A range of tissues was used in this study obtained with consents and approvals as described previously [3], including Local Research Ethics Committee, Royal National Orthopaedic Hospital, Stanmore, UK, Material Transfer Agreement, and Netherlands Brain Bank. Specimens were snap frozen in liquid nitrogen and stored at −70°C until use or immersed in Zamboni’s fixative (2% w/v formalin, 0.1 M phosphate, and 15% v/v saturated picric acid) for 2 h and stored in phosphate buffered saline (PBS) containing 15% sucrose, 0.01% azide.

#### Immunohistology

Tissues were supported in optimum cutting tissue (OCT) medium (RA Lamb Ltd, Eastbourne, UK). Tissue sections (15 µm thick) were collected onto coated glass slides and post-fixed in 4% w/v paraformaldehyde in 0.15 M phosphate buffered saline (PBS) for 30 min (for frozen section only). Endogenous peroxidase was blocked by incubation in methanol containing 0.3% w/v hydrogen peroxide for 30 min. After rehydration with PBS buffer, sections were incubated overnight with primary antibody using a range of dilutions (Table 1).

Sites of primary antibody attachment were revealed using nickel-enhanced, avidin–biotin peroxidase (ABC-Vector Laboratories, Peterborough, UK) as previously described [38]. Sections were counter-stained for nuclei in 0.1% w/v aqueous neutral red, dehydrated and mounted in xylene-based mountant (DPX; BDH/Merck, Poole, UK), prior to photomicrography.

### Analysis of data

#### DRG

Antibody-immunoreactive, nucleated neurons in sensory ganglia (DRG) were counted and their diameter assessed using a calibrated microscope eyepiece graticule and expressed as % total; image analysis (% area) of nerve sections has been described previously [3].

### ELISA for AngII, AngIII and Ang-(1-7)

#### AngII ELISA

Frozen tissue samples of control normal nerve (n = 31), injured nerves (n = 7), and painful neuromas (n = 12), were weighed and extracted in boiling 0.5 M acetic acid for 10 min. Extracts were then concentrated and de-salted by applying 0.2 ml extract to an activated Sep-Pak column, washed and desalted with water containing 0.1% TFA. Bound peptides were eluted from the columns using 1 ml of 80% acetonitrile containing 0.1% TFA. The eluate was evaporated to dryness using a speed-vac overnight. Each vial was reconstituted in 1× assay buffer supplied by the manufacturer (see below).

A human specific AngII/AngIII immunoassay kit (EK-002-12, reacting 100% to AngII and AngIII, Phoenix Pharmaceuticals, Burlingame, California, USA) was used according to the manufacturer’s instructions (but note AngIII specific immunoassay kit described below showed undetectable levels of AngIII in these nerve extracts). AngII standard (0.04–25 ng/ml) and specimens (50 µl) were added in duplicate and the mean used for subsequent analysis. The absorbance at 450 nm in each well was measured using a plate reader. Standard curve was plotted using a log scale and the concentration of human AngII in each specimen determined using Excel software.

In order to determine the recovery of AngII after extracting in boiling in acetic acid and concentrating using a Sep PaK, 200 µl of standard (at 1 nmol/ml) was added to 800 µl of boiling acetic acid for a further 10 min, and after cooling 200 µl was applied to Sep Pak and dried overnight as above. This was reconstituted with 200 µl of assay buffer and 50 µl used for assay in duplicate.

#### AngIII

A human specific AngIII immunoassay kit (USCN Life Sciences ELISA kit, E92312Hu, Diagenics Limited, Milton Keynes, England) that reacts 100% to AngIII and with no significant cross-reactivity with analogues, was used according to the manufacturer’s instructions. AngIII standard (6.17–500 pg/ml) and specimens (50 µl) were added in quadruplicate and the mean used for subsequent analysis. The absorbance at 450 nm in each well was measured using a plate reader. Standard curve was plotted and the concentration of human AngIII in each specimen determined using Excel.

#### Ang-1-7

A human specific Ang-1-7 immunoassay kit (USCN Life Sciences ELISA kit, E86085Hu, Diagenics Limited, Milton Keynes, England), reacting 100% to Ang-(1-7) and no significant cross-reactivity with analogues, was used according to the manufacturer’s instructions. Ang-(1-7) standard (12.35–1,000 pg/ml) and specimens (50 µl) were added in quadruplicate and the mean used for subsequent analysis. The absorbance at 450 nm in each well was measured using a plate reader. Standard curve was plotted and the concentration of human Ang-(1-7) in each specimen determined using Excel.

### In vitro studies

#### Preparation of hDRG neurons

hDRG were obtained from five patients with brachial plexus avulsion undergoing nerve repair surgery, excised as a necessary part of surgical repair i.e. redundant tissue; ganglia were enzyme digested, mechanically dissociated and plated on collagen and laminin coated MatTek dishes (MatTek Corp USA), in Ham’s F12 medium containing 10% HIFCS (heat inactivated fetal calf serum), penicillin/streptomycin (100 μg/ml each), NTFs (NGF 100 ng/ml, GDNF and NT3 50 ng/ml each), as previously described [3], for 48 h at 37°C before further studies.

#### Immunofluorescence for AngII, AT2R, TRPV1 in hDRG neurons

Cultures were fixed in 4% PFA for 30 min, permeabilised with chilled methanol (−20°C, 3 min), rehydrated in PBS and incubated in primary antibodies to goat anti AT2R (SC48452, 1:200, Santa cruz), rabbit anti TRPV1 (1:500, GSK), or goat anti β tubulin (1:200 Abcam), mouse anti AngII (Serotec 1:200), rabbit anti PGP9.5 (1:500, Ultraclone, RA95/06), mouse anti Gap43 (1:200, Sigma, UK), and visualised with secondary antibodies (1:200 each, Life Technologies, U.K.), donkey anti mouse (Alexa 488), donkey anti rabbit (Alexa 350), and donkey anti goat (Alexa 546), incubated for 1 h at room temperature, and mounted on glass slides in Citifluor mounting medium with the antifade agent DABCO (Sigma, UK). TIFF images were acquired using widefield fluorescence optics with an upright Olympus microscope BX43, and cooled CCD camera (Coolsnap), using uv illumination, and Cellsens software (Olympus, Japan). Cell counts were obtained for neurons expressing AT2R, AngII and TRPV1, and neuronal diameters were measured using Cellsens software.

#### Calcium imaging in human DRG neurons

48 h after plating, hDRG neurons were loaded with 2 μM Fura2 AM, and responses to capsaicin, AngII, and EMA1087 (C21), were imaged as before [3]. In each experiment, capsaicin sensitive neurons were identified with a brief 200 nM capsaicin stimulus (30 s), and washout of medium. After a rest period of 40 min, a second capsaicin stimulus, of 1 μM was applied with or without drugs. The effects of the kinase inhibitor staurosporine, MAPK inhibitor PD98059 and TrkA inhibitor GW441657 on AngII mediated sensitization were determined by adding either one of the inhibitors 10 min prior to adding AngII, followed 10 min later, by the second capsaicin stimulus. The second capsaicin response was normalised to the first, for calculating the percent response, and the average calculated for each group.

#### Preparation of rDRG neurons

Bilateral DRG from all levels of 6 adult female Wistar rats were isolated and plated as before [3], in collagen/laminin coated glass bottom plastic petri dishes (MatTek, USA), at 1,000 neurons/dish, in BSF2 medium without NTFs for pp38 and pp42 immunofluorescence and neurite length assay, and with NTFs for calcium imaging, at 37°C. Calcium imaging studies in rat DRG neurons were carried out as for human neurons above.

#### Immunofluorescence for pp38 and pp42 expression in AngII and EMA401 treated adult rat DRG neurons

48 h after plating, duplicate dishes were treated with AngII (10 nM), AngII + EMA401 (10 and 100 nM respectively), 100 nM EMA401, NGF (100 ng/ml) or vehicle treated (control 0), for 30 min at 37°C, then fixed with 4% PFA for 30 min for immunostaining.

Following fixation, and 3 min permeabilisation with methanol at −20°C, the neurons were incubated in rabbit polyclonal antibody to phospho-p38MAPK pThr180 (1:200, PA1-14304, Thermo Scientific, USA), or rabbit monoclonal antibody to phospho –p44/42 (ERK 1/2, 1:200, 4370S New England Biolabs, UK), for 1 h at room temperature, visualised with secondary antibody Alexa fluor 546 goat anti rabbit (1:200, Life Technologies, USA), incubated for 45 min, and mounted in glycerol containing antifade agent DABCO (Sigma UK) on glass slides. TIFF fluorescence images were acquired with an upright Olympus BX43 widefield fluorescence microscope with the same acquisition settings for all groups, using Cellsens software (Olympus, Japan). Signal intensity was measured after background substraction, from at least 120 neurons in each group, from at least three experiments using Image J software (N.I.H. USA), and normalised to controls.

#### Effect on neurite length

rDRG neurons were prepared as above in BSF2 medium, and 48 h later duplicate dishes were treated with AngII (10 nM), NTFs (NGF 100 ng/ml, GDNF and NT3 50 ng/ml each), 10 nM EMA1087 (Compound **21**) or vehicle treated, for 48 h at 37°C. Cultures were fixed and immunostained with mouse Gap43 (1:200, Sigma, UK), visualised with FITC conjugated Alexa Fluor goat anti mouse (1:200, Life Technologies), for 45 min each, and mounted on glass slides. TIFF images were acquired with an Olympus upright microscope as above. The longest neurite lengths were measured for calculating the average from approximately 50 neurons in each group and normalised to vehicle treated controls.

Student’s *t* test was used to compare groups, and P < 0.05 was considered statistically significant.

## Abbreviations

AngII: angiotensin II
AT_2_R: angiotensin II subtype 2 receptor
AngIII: angiotensin III
ELISA: enzyme linked immunosorbent assay
TRPV1: transient receptor potential vanilloid subtype 1
FITC: fluorescene isothiocyanate
ERK: extracellular signal related kinase
NGF: nerve growth factor
GDNF: glial cell-line derived neurotrophic factor
NT3: neurotrophin 3
MAPK: mitogen activated protein kinase
PFA: paraformaldehyde
DRG: dorsal root ganglion
hDRG: human dorsal root ganglion
rDRG: rat dorsal root ganglion
